# Optimization of Hydrolysis Conditions, Isolation, and Identification of Biologically Active Peptides Derived from *Acheta domesticus* for Antioxidant and Collagenase Inhibition

**DOI:** 10.3390/antiox13030367

**Published:** 2024-03-18

**Authors:** Kankanit Yeerong, Panuwan Chantawannakul, Songyot Anuchapreeda, Sutee Wangtueai, Wantida Chaiyana

**Affiliations:** 1 Department of Pharmaceutical Sciences, Faculty of Pharmacy, Chiang Mai University, Chiang Mai 50200, Thailand; kankanit_yeerong@cmu.ac.th; 2 Research Center of Deep Technology in Beekeeping and Bee Products for Sustainable Development Goals: SMART BEE SDGs, Chiang Mai University, Chiang Mai 50200, Thailand; panuwan.c@cmu.ac.th; 3 Division of Clinical Microscopy, Department of Medical Technology, Faculty of Associated Medical Sciences, Chiang Mai University, Chiang Mai 50200, Thailand; songyot.anuch@cmu.ac.th; 4 Center of Excellence in Pharmaceutical Nanotechnology, Faculty of Pharmacy, Chiang Mai University, Chiang Mai 50200, Thailand; 5 College of Maritime Studies and Management, Chiang Mai University, Samut Sakhon 74000, Thailand; sutee.w@cmu.ac.th; 6 Multidisciplinary and Interdisciplinary School, Chiang Mai University, Chiang Mai 50200, Thailand; 7 Innovation Center for Holistic Health, Nutraceuticals, and Cosmeceuticals, Faculty of Pharmacy, Chiang Mai University, Chiang Mai 50200, Thailand

**Keywords:** house cricket, protein hydrolysate, anti-skin aging, enzymatic hydrolysis, peptides

## Abstract

The study aimed to optimize hydrolysis conditions and isolate and identify bioactive peptides with anti-skin aging effects from *Acheta domesticus* (house cricket). *A. domesticus* proteins underwent hydrolysis using Alcalase^®^ and optimized conditions using response surface methodology through a face-centered central composite design. Variable controls (enzyme–substrate concentration (E/S), time, and temperature) were assessed for their impact on activities against collagenase, 2,2-diphenyl-1-picrylhydrazyl radical (DPPH^●^), and degree of hydrolysis of protein hydrolysate (PH). PH was also investigated for composition, anti-skin aging, and anti-inflammatory effects. Amino acid sequences with potent anti-skin aging activity were isolated and identified using ultrafiltration, gel filtration chromatography, and liquid chromatography coupled with tandem mass spectrometry, employing de novo sequencing. Optimal conditions for producing PH with maximum anti-skin aging activity were an E/S concentration of 2.1% (*w*/*w*), 227 min, and 61.5 °C. Glutamic acid was a predominant amino acid and PH exhibited a molecular weight below 15 kDa. Additionally, PH displayed significant activities against collagenase, hyaluronidase, DPPH^●^, lipid peroxidation, and NF-κB-mediated inflammation (*p* < 0.05). Three novel anti-skin aging peptides were identified—Ala-Val-Thr-Lys-Ala-Asp-Pro-Tyr-Thr-Asp-Gln, Thr-Val-Met-Glu-Leu-Asn-Asp-Leu-Val-Lys-Ala-Phe, and Val-Pro-Leu-Leu-Glu-Pro-Trp—exhibiting the most potent collagenase and DPPH^●^ inhibition. Therefore, this study proposed that PH, produced with Alcalase^®^ under optimal conditions, emerges as a promising substance with potent anti-skin aging activity for the cosmeceutical industry.

## 1. Introduction

Nowadays, societies worldwide have shifted into aging societies due to the simultaneous rise in the number and life expectancies of older individuals [1]. Skin aging emerges as one of the most distinctive physical issues associated with the aging process. This phenomenon hinders both the visual appearance and functional aspects of the skin, revealing the presence of deep wrinkles, rough texture, progressive dryness, sagging, and complex pigmentation [2,3]. Furthermore, this process can increase the risk of skin diseases, including skin cancer [4]. Thus, skin aging not only affects a person’s mental well-being but also influences their overall quality of life [5]. Skin aging is typically categorized into two types: intrinsic and extrinsic. Intrinsic aging is linked to genetic factors, whereas extrinsic aging is influenced by external environmental factors such as air pollution, cigarette smoking, and notably, ultraviolet (UV) irradiation [6]. In this context, UV exposure could induce oxidative stress, leading to a reduction in the activity of the skin’s antioxidant defense enzyme system and thereby increasing susceptibility to oxidative damage [7]. Furthermore, UV-induced oxidative damage activates multiple signaling pathways, including the mitogen-activated protein kinase and nuclear factor-kappa B (NF-κB) pathways. This activation results in the production of matrix metalloproteinases (MMPs). MMP-1 belongs to the collagenases subclass and is primarily released by epidermal keratinocytes and dermal fibroblasts in skin layers [8,9,10]. The function of MMP-1 involves the cleaving of mesenchymal collagen, leading to the degradation of type I and type III collagen at a precise location in their triple helix [11,12]. This process ultimately induces damage to the extracellular matrix (ECM), playing a role in the development of wrinkles and a decline in skin elasticity [13]. Furthermore, skin aging is fundamentally linked to the loss of moisture in the skin [14]. Hyaluronic acid (HA) is a key molecule in skin hydration, possessing a unique ability to retain water [15]. However, this crucial molecule can be degraded by hyaluronidase (HAase), which hydrolyzes the β-1,4-glycosidic bonds of hyaluronic acid, leading to a reduction in skin moisture [16]. Hence, the exploration of inhibitors for these molecules is crucial and can serve as a vital approach to the renewal and recovery of aging skin.

Peptides, defined as short chains consisting of two to twenty amino acids linked by peptide bonds and possessing a molecular weight (MW) of less than 10 kDa [17,18], have attracted significant attention from scientists as notable bioactive compounds [19]. These compounds manifest assorted biological effects, focusing activity against skin aging, such as antioxidant [20], anti-aging [21], moisturizing [22], collagen-stimulating [23], and wound healing effects [24]. Moreover, peptides are valued for their high safety profiles, hypoallergenicity, and cost-effective production methods, supported by evidence from both in vitro and in vivo studies, as well as clinical trial outcomes [25,26,27]. Bioactive peptides can be sourced from natural origins such as plants, animals, and marine sources; however, these peptides lack bioactivity when they remain bound within the intact protein structure [28]. The activation of bioactive peptides is facilitated through processes, for instance, involving endogenous enzymes, using exogenous enzymes (commercial preparations), and microbial fermentation [29,30]. Enzymatic hydrolysis is widely used to improve and modify protein functionality from a wide range of protein sources to release bioactive fragments [31]. Therefore, the exploration of new sources for producing bioactive peptides and the processes to liberate bioactive fragments represents a challenging endeavor.

In recent years, there has been a growing interest in exploring insect proteins as potential sources of bioactive agents [32]. The house cricket, scientifically known as *Acheta domesticus* and belonging to the Orthoptera order and Gryllidae family [33], stands out as a fascinating option for protein sourcing due to its high protein content [34]. Numerous studies have highlighted the bioactive properties of cricket preparations, including extracts, protein isolates, and protein hydrolysates (PH), showcasing diverse biological effects, including antioxidant activity [35], the ability to inhibit enzymes associated with hypertension [36] and type-2 diabetes [37], and anti-inflammatory effects [38]. Furthermore, several studies have suggested that the application of enzymatic treatment not only improves bioactivity properties but also diminishes the allergenicity of proteins derived from crickets [27,39]. The study by Hall et al. (2018) reported that cricket proteins following hydrolysis with the Alcalase^®^ enzyme exhibited no reactivity with human shrimp-allergic sera [27]. However, there is currently no study directly employing *A. domesticus* to produce PH for anti-skin aging properties. Our previous study revealed that aqueous extracts from *A. domesticus* demonstrated robust activities against enzymes linked to skin aging and oxidative stress and proposed that the proteins could potentially be responsible for these observed activities [40]. Consequently, the utilization of enzymatic hydrolysis may further enhance the effectiveness against skin aging and the safety of *A. domesticus* protein. Therefore, the objective of this study was to optimize hydrolysis conditions and isolate and identify the bioactive peptides exhibiting the most potent anti-skin aging effects from the PH of *A. domesticus*. The results of this study may serve as evidence supporting the utilization of PH from *A. domesticus* as cosmeceutical compounds for addressing skin aging.

## 2. Materials and Methods

### 2.1. Insect Materials

**Figure 1:**
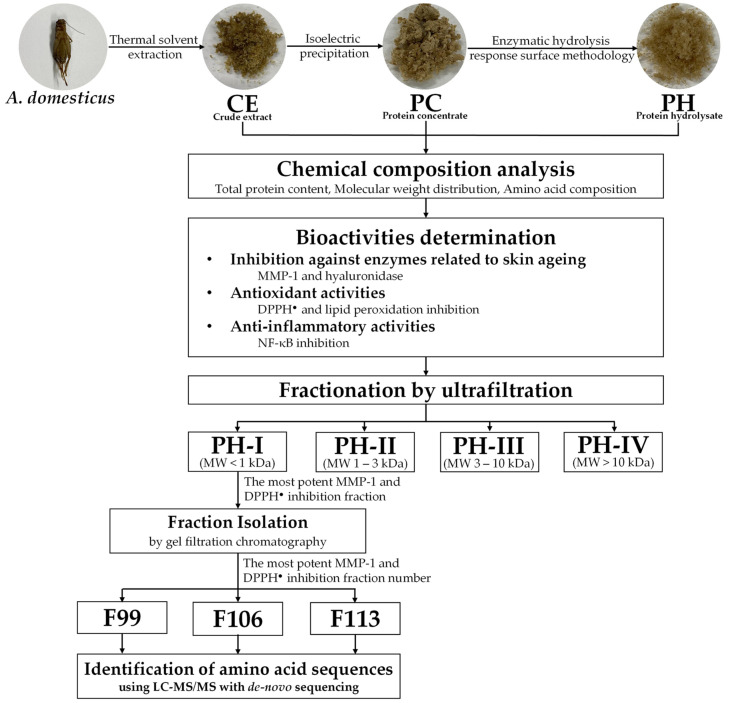
Schematic representation of the analytical procedure for obtaining and characterizing crude extract (CE), protein concentrate (PC), and protein hydrolysate (PH), including PH-I, PH-II, PH-III, and PH-IV fractions. The diagram illustrates the sequential steps involved in extraction, concentration, and fractionation, facilitating the assessment of anti-skin aging properties.

Frozen *A. domesticus* was procured from the Ruamchok market in Chiang Mai, Thailand. Thawing was conducted at room temperature overnight, followed by drying in a hot air oven (Memmert, Schwabach, Germany) set to 45 °C for 24 h. The dried *A. domesticus* was then processed into a fine powder using a Moulinex™ blender (model LM2070BD, Moulinex SA, Bagnolet, France). Subsequently, *A. domesticus* fine powder was hermetically sealed in a container and stored at (2 to 4) °C for further analysis, as shown in Figure 1.

### 2.2. Chemical Materials

Alcalase^®^ enzyme from *Bacillius licheniformis* was purchased from EMD Millipore in Darmstadt, Germany. 3-(4,5-Dimethylthiazol-2-yl)-2,5-diphenyltetrazolium bromide (MTT), quercetin, gallic acid, collagenase from *Clostridium histolyticum*, *N*-[3-(2-furyl)acryloyl]-Leu-Gly-Pro-Ala (FALGPA), hyaluronidase from bovine testes, hyaluronic acid sodium salt from *Streptococcus equi*, picrylsulfonic acid solution (TNBS), sodium phosphate monobasic monohydrate (NaH_2_PO_4_•H_2_O), L-leucine, oleanolic acid, 1,1-diphenyl-2-picrylhydrazyl (DPPH), linoleic acid, L-ascorbic acid (purity ≥ 99.0%), sodium thiocyanate, and ferric chloride (FeCl_3_) were purchased from Sigma Aldrich in St. Louis, MO, USA. Disodium hydrogen phosphate (Na_2_HPO_4_), potassium dihydrogen phosphate (KH_2_PO_4_), potassium chloride (KCl), sodium carbonate (Na_2_CO_3_), and sodium chloride (NaCl) were purchased from Fisher Chemicals in Loughborough, UK. Trichloroacetic acid (TCA), bicinchoninic acid (BCA) protein assay kit, and Luminata™ Western HRP chemiluminescence substrates were purchased from Merck KGaA in Darmstadt, Germany. Sodium hydroxide (NaOH), dimethyl sulfoxide (DMSO), hydrochloric acid (HCl), petroleum ether, methanol, acetic acid, and ethanol were analytical-grade and purchased from RCI Labscan Co., Ltd. in Bangkok, Thailand. RPMI-1640 medium, Dulbecco’s modified eagle’s medium (DMEM), L-glutamine, and penicillin-streptomycin were purchased from GIBCO Invitrogen in Grand Island, NY, USA. Fetal bovine serum (FBS) was purchased from Biochrom AG in Berlin, Germany. Boric acid (H_3_BO_3_) and sodium tetraborate dodecahydrate (Na_2_B_4_O_7_•10H_2_0) were purchased from QRëC in the Republic of New Zealand (Auckland, New Zealand). Sodium sulfate anhydrous (Na_2_SO_4_) was purchased from Loba Chemie in Mumbai, India. Laemmli sample buffer, 2-mercaptoethanol, 10× Tris/Glycine/SDS, resolving gel buffer, restacking gel buffer, and Coomassie brilliant blue R-250 were purchased from Bio-Rad Laboratories in Hercules, CA, USA. Scientific™ Spectra™ Multicolor Broad Range Protein Ladder and RIPA buffer were purchased from Thermo Fisher Scientific in Waltham, MA, USA. Rabbit monoclonal anti-NF-κB IgG and rabbit polyclonal anti-human glyceraldehyde 3-phosphate dehydrogenase (GAPDH) IgG were purchased from Cell Signaling Technology in MA, USA. HRP-conjugated goat anti-rabbit IgG was purchased from Promega in Madison, WI, USA.

### 2.3. Preparation of Crude A. domesticus Extract (CE) by Thermal Solvent Extraction

CE was produced through thermal solvent extraction, following the methodology outlined in our earlier study [40]. Briefly, finely ground *A. domesticus* powder was extracted with deionized (DI) water for 3 h at 45 °C using a water bath (Memmert, Schwabach, Germany). Consequently, the obtained mixture was subjected to centrifugation at 3000× *g* for 15 min using a laboratory centrifuge (MPW-352R, MPW Med. Instruments, Warsaw, Poland) and then the supernatant was collected and filtered through Whatman No. 1 filter papers. Subsequently, the filtered supernatant was frozen and dried using a freeze dryer (Beta 2–8 LD-plus, Martin Christ Gefriertrocknungsanlagen GmbH, Osterode am Harz, Germany). CE was kept at −20 °C until further experiments.

### 2.4. Preparation of Protein Concentrate (PC) by Isoelectric Precipitation

PC was generated using the isoelectric precipitation technique, following the methodology of Quinteros et al. (2022) with some modifications [38]. Briefly, CE was partially defatted by soaking in petroleum ether in a ratio of 1:10 for 4 h. Then the supernatant was removed, and the residue was left to stand in a fume hood overnight, allowing the solvent to evaporate. The defatted CE was redissolved with DI water. Consequently, the pH of the mixture was then raised to 11.0 using 1 M NaOH solution, and the mixture was stirred continuously using a magnetic stirrer for 1 h. Afterward, the resulting mixture was subjected to centrifugation at 3000× *g* for 15 min. The supernatant was carefully collected, and the pH was readjusted to 4.5 using 1 M HCl solution. Subsequently, the mixture was centrifuged once more at 3000× *g* for 15 min. The pellet was collected and subjected to drying using a freeze dryer, resulting in PC formation.

### 2.5. Optimization of PH Preparation by Enzymatic Hydrolysis

The experimental design, data analysis, and optimization of the enzymatic hydrolysis conditions to produce the PH were performed using Design Expert version 13 software from Stat-Ease, Inc. (Minneapolis, MN, USA). Response surface methodology (RSM) with a 3-factor, 3-level based on face-centered central composite design (CCD) was used in the present study [41]. Prior to the enzymatic hydrolysis process, PC was redispersed in DI water and adjusted to pH 7.5 using 1 M NaOH solution. The enzymatic hydrolysis was carried out using Alcalase^®^. Three controllable variables were chosen for optimization: enzyme–substrate concentration ((1 to 3) %*w*/*v*, X_1_), hydrolysis time ((120 to 300) min, X_2_), and temperature ((55 to 65) °C, X_3_). Twenty hydrolysis conditions were generated by varying these three parameters and their corresponding levels, as detailed in Table 1.

To stop the hydrolysis reaction, the enzyme was deactivated by heating at 90 °C for 20 min in a water bath. After being subjected to centrifugation at 8000× *g* for 15 min, the resulting supernatant was collected and subjected to freeze-drying, yielding PH, which was stored at −20 °C for further analysis. Three responses, including MMP-1 inhibition activity (%, Y_1_), DPPH radical (DPPH^●^) scavenging ability (%, Y_2_), and degree of hydrolysis (%, Y_3_), were determined as the response variables. A polynomial regression equation for dependent variables was established, represented by Equation (1):

Y = β_0_ + β_1_X_1_ + β_2_X_2_ + β_3_X_3_ + β_11_X_1_^2^ + β_22_X_2_^2^ + β_33_X_3_^2^ + β_12_X_1_X_2_ + β_13_X_1_X_3_ + β_23_X_2_X_3_ (1)

where Y is a predicted response; X_1_, X_2_, and X_3_ are independent variables; β_0_ is an offset term; β_1_, β_2_, and β_3_ are linear terms; β_11_, β_22_, and β_33_ are quadratic terms; β_12_, β_13_, and β_23_ are interaction terms. Each of the experiments was performed independently in triplicate.

### 2.6. Degree of Hydrolysis Determination

PH was assessed for degree of hydrolysis following a methodology detailed in the previous study of Hall et al. (2017) [27]. The content of α-amino acids was quantified based on the calibration curve using L-leucine as a reference. The hydrolysis degree was calculated following Equation (2):

% Degree of hydrolysis = (α_t_ − α_0_)/(α_max_ − α_0_) × 100 (2)

where α_t_ represents the L-leucine equivalence amount obtained from PH at a given time point, α_0_ represents the L-leucine equivalence amount from PC, and α_max_ represents the L-leucine equivalence amount from PH, obtained through full hydrolysis with 12 N HCl at 100 °C over 24 h. Each of the experiments was performed independently in triplicate.

### 2.7. Determination of Inhibitory Activities against Enzymes Related to Skin Aging

#### 2.7.1. MMP-1 Inhibitory Activity

CE, PC, and PH were investigated for MMP-1 inhibitory activity using a spectrophotometry assay based on the enzymatic reaction method following Thring et al. (2009) with some modifications [42]. The results were presented as a percentage of MMP-1 inhibition, which was calculated using Equation (3):

% MMP-1 Inhibition = [(A − B)/A] × 100(3)

where A is the reaction rate of the mixture containing MMP-1, tricine buffer, and FALGPA solution, and B is the reaction rate of the mixture containing the sample, MMP-1, tricine buffer, and FALGPA solution. Oleanolic acid was used as a positive control. The results were also presented as IC_50_ values, representing the concentration of the sample suppressing MMP-1 activity by 50%. Each of the experiments was performed independently in triplicate.

#### 2.7.2. Hyaluronidase Inhibitory Activity

CE, PC, and PH were investigated for hyaluronidase inhibition by a spectrophotometry assay based on the enzymatic reaction method following a previous study by Jiamphun and Chaiyana (2022) [43]. The results were presented as IC_50_ values, representing the concentration of the sample suppressing hyaluronidase activity by 50%. Oleanolic acid was used as a positive control. Each of the experiments was performed independently in triplicate.

### 2.8. Determination of Antioxidant Activities

#### 2.8.1. DPPH Assay

CE, PC, and PH were determined for DPPH^●^ scavenging activity using the DPPH assay following the study of Somwongin et al. (2023) [44]. DPPH^●^ scavenging capabilities were reported as a percentage of DPPH^●^ inhibition, which was calculated using Equation (4):

% DPPH^●^ Inhibition = [(A − B)/A] × 100 (4)

where A is the absorbance of the mixture solution containing DPPH^●^ solution and solvent and B is the absorbance of the mixture solution containing sample and DPPH^●^ solution. Ascorbic acid was used as a positive control. The results are also presented as IC_50_ values, representing the concentration of the sample suppressing DPPH^●^ by 50%. Each of the experiments was performed independently in triplicate.

#### 2.8.2. Inhibition of Lipid Peroxidation by Ferric Thiocyanate (FTC) Assay

CE, PC, and PH were investigated for activity against lipid peroxidation by FTC assay following the study of Somwongin et al. (2023) [44]. The results were presented as IC_50_ values, representing the concentration of the sample suppressing lipid peroxidation by 50%. Trolox was used as a positive control. Each of the experiments was performed independently in triplicate.

### 2.9. Chemical Composition Analysis

#### 2.9.1. Total Protein Content

CE, PC, and PH were investigated for total protein content using the BCA assay, according to the study of Yeerong et al. (2021) [40]. The results were reported as percentages of protein content compared to the dry weight of the sample. Each of the experiments was performed independently in triplicate.

#### 2.9.2. Protein MW Distribution Profile

CE, PC, and PH were inspected for MW distribution profiles using sodium dodecyl sulfate–polyacrylamide gel electrophoresis (SDS-PAGE) adapted from Montowska et al. (2019) [45]. In brief, after 10 μL of each sample (10 mg/mL) was mixed with 10 μL of Laemmli sample buffer and 2-mercaptoethanol (reducing agent), the resulting mixture was then heated at 90 °C for 5 min and loaded into each well of 12% SDS-PAGE gel, which was subjected to the electrophoresis cell with a constant current of 100 V. Spectra™ multicolor broad range protein ladder was utilized as a MW protein standard. Consequently, the gel was washed using the solution containing 10% *v/v* acetic acid with 40% *v*/*v* methanol and then stained with 0.025% *w*/*v* Coomassie brilliant blue R-250 for 1 h. Afterward, the gels were de-stained using 10% *v*/*v* acetic acid with continuous agitation until the background was clear. The SDS-PAGE gel image was captured using a gel documentation system and protein bands were analyzed for their MW using Image Lab software (version 6.1, Gel Doc^TM^ EZ Imager, Bio-Rad Laboratories, Hercules, CA, USA). Each of the experiments was performed independently in duplicate.

#### 2.9.3. Amino Acid Composition

PH was examined for amino acid profile according to the method of Yin et al. (2010) using ion exchange chromatography followed by post-column ninhydrin derivatization by an amino acid analyzer (Model L-8900, Hitachi High-Tech Corp., Hitachi, Japan) [46]. To establish calibration curves, a standard amino acid mixture was employed. The curve was constructed by plotting the peak area of individual amino acids against their predetermined concentrations. The amino acid content of PH was subsequently determined and expressed in units of grams per 100 g of PH.

### 2.10. Determination of Cytotoxicity by MTT Assay

CE, PC, and PH were observed for cytotoxicity on immortalized human keratinocyte (HaCaT) cells using the MTT cell proliferation assay following the study of Panyajai et al. (2024) [47].

The results were expressed as IC_20_ values, representing the concentration of the sample suppressing cell viability by 20%. GraphPad/Prism program version 8.0 (GraphPad Software Inc., La Jolla, CA, USA) was used to plot the dose–response relationship. Each of the experiments was performed independently in triplicate.

### 2.11. Determination of Anti-Inflammatory Activity

CE, PC, and PH at their concentrations of IC_20_ on HaCaT cells were observed for NF-κB inhibitory activity using Western blotting according to the method of Neimkhum et al. (2021) [48]. For protein band detection, Luminata™ Forte Western HRP substrate was applied to the membrane and then exposed to X-ray film (Fine Med Company, Bangkok, Thailand). Densitometry was analyzed using Quantity One 1-D Analysis software (version 4.6.6, Bio-Rad Laboratories, Hercules, CA, USA). The density values of NF-κB bands were normalized to GAPDH bands. The results were expressed as the percentage of NF-κB inhibition. Dexamethasone was used as a positive control. Each of the experiments was performed independently in triplicate.

### 2.12. Fractionation and Isolation of Bioactive Peptides from PH

#### 2.12.1. Fractionation of PH by Ultrafiltration

PH was fractionated using an Amicon^®^ stirred cell (Merck KGaA, Darmstadt, Germany) with cellulose ultrafiltration membranes following the method of Doungapai et al. (2022) [49]. A solution of PH (50 mg/mL) was loaded in the Amicon^®^ stirred cell and fractionated through a series of ultrafiltration membranes with nominal MW cut-off values of 10, 3, and 1 kDa. Four fractions of <1, 1–3, 3–10, and >10 kDa were obtained as PH-I, PH-II, PH-III, and PH IV, respectively. Each of these fractions was subsequently dried using a freeze dryer. Each fraction was assessed for MMP-1 inhibitory and DPPH^●^ scavenging activities. Oleanolic and ascorbic acid were used for standard curve creation of MMP-1 inhibitory and DPPH^●^ scavenging capabilities, respectively. The results were expressed as microgram oleanolic equivalent per milligram protein (µg OAE/mg protein) and microgram ascorbic acid equivalent per milligram protein (µg AAE/mg protein), respectively. Each of the experiments was performed independently in triplicate.

#### 2.12.2. Fraction Isolation by Gel Filtration Chromatography

The fraction with the most potent MMP-1 inhibitory and DPPH^●^ scavenging activities was further isolated using gel filtration chromatography [49]. The dried fraction was reconstituted in DI water and the resulting mixture was subjected to isolate using a Sephadex G-25 column measuring 2.6 × 70 cm (GE Healthcare Bio-Science AB in Uppsala, Sweden) with an MW separation range of 1000 to 5000. The separation process was carried out using a BioLogic low-pressure chromatography system (Bio-Rad Laboratories, Hercules, CA, USA) connected to a Biofrac fraction collector. The elution was performed with DI water at a flow rate of 0.5 mL/min. All the collected fractions, each consisting of 3 mL, were analyzed for their absorbance at 220 and 280 nm and protein concentration using the BCA assay. Consequently, each fraction was determined for MMP-1 inhibitory and DPPH^●^ scavenging activities. The standard compounds with known MW, including blue dextran (2,000,000 Da), insulin B (3495.9 Da), vitamin B12 (1355.5 Da), glycine-tyrosine (238.2 Da), and tyrosine (181 Da), were used for the construction of standard curves for the estimations of the MW of peptides in each fraction.

### 2.13. Identification of Bioactive Peptide from PH

The fraction with the most potent MMP-1 inhibitory and DPPH^●^ scavenging activities was subjected to sequencing and accurate determination of their molecular masses using LC-MS/MS with de novo sequencing, following the method of Krobthong and Yingchutrakul (2020) with minor modifications [50]. In brief, 3 mg of each fraction underwent loading onto a pre-equilibrated Sep-Pak C18 column (Waters, Milford, MA, USA) and was subsequently eluted using a gradient ranging from (40 to 35)% *v*/*v* acetonitrile in water. The eluted material was then concentrated using a SpeedVac vacuum concentrator (Thermo Scientific Inc., Franklin, MA, USA). After concentration, the peptide fraction was reconstituted in a solution containing 0.1% *v*/*v* formic acid and subjected to Orbitrap HF LC-MS/MS analysis (Thermo Scientific Inc., Franklin, MA, USA). The LC-MS/MS separation involved a 120 min gradient of mobile phase A (0.1% *v*/*v* formic acid in water) and mobile phase B (0.1% *v*/*v* formic acid in 80% *v*/*v* acetonitrile) at a flow rate of 250 mL/min. Mass spectra were acquired in positive mode within an m/z range of 600 to 2200, employing a data-dependent acquisition mode. Spectra were recorded for ions carrying + 2, + 3, + 4, and + 5 charge states and subsequently analyzed using PeakX Studio 10.0 software (Bioinformatics Solution Inc., Waterloo, ON, Canada). De novo peptide sequencing was conducted for the peptide ion with the highest intensity, and the acceptability of de novo peptide sequences was determined based on a filter of ≥95% average local confidence (ALC).

### 2.14. Statistical Analysis

The statistical analyses for RSM were performed using Design-Expert software (version 13.0; Stat-Ease Inc., Minneapolis, MN, USA). This included analysis of variance (ANOVA), determination of regression coefficients (R^2^), testing for lack of fit, optimizing enzymatic hydrolysis conditions, and the generation of three-dimensional graphs. Data were presented as mean ± standard deviation (SD) based on three independent experiments. For data statistical evaluation, a one-way ANOVA was conducted, followed by a post hoc Tukey test, utilizing GraphPad Prism version 8.0 (GraphPad Software Inc. in La Jolla, CA, USA). Statistical significance was established at a *p*-value less than 0.05.

## 3. Results and Discussions

### 3.1. Optimized Hydrolysis Conditions for PH Generation

RSM was used to investigate the combination effects of independent variables on the responses and optimize the enzymatic hydrolysis conditions for PH preparation. Overall, 20 runs of hydrolysis experiments were carried out based on central composite design (CCD) and six central points with three independent factors, including enzyme–substrate concentration (E/S) ((1 to 3)% *w*/*w*, X_1_), hydrolysis time ((120 to 300) min, X_2_), and temperature ((55 to 65) °C, X_3_) on three responses, comprising MMP-1 inhibition activity (%, Y_1_), DPPH^●^ scavenging activity (%, Y_2_), and degree of hydrolysis (%, Y_3_). An ANOVA was used to evaluate the statistical significance of the models. The experimental results of each run under the specified conditions are detailed in Table 2. Additionally, the polynomial regression equation was used to fit the relationship of variable factors with the corresponding response values.

#### 3.1.1. MMP-1 Inhibitory Activity

The activities against MMP-1 of PH ranged from (48.3 to 61.3)% under different hydrolysis conditions. This variability demonstrated a quadratic relationship between the anti-MMP1 and hydrolysis variables in a statistically significant model (*p* < 0.0001), affirming the robustness and appropriateness of the model. The mathematical model, derived through multiple regression analysis, was expressed as a second-order polynomial equation in coded form, denoted as Equation (5).

Y_1_ = 59.0 + 0.2X_1_ − 0.1X_3_ + 1.7X_1_X_2_ + 0.1X_1_X_3_ − 2.2X_2_X_3_ − 2.0X_1_^2^ − 3.7X_2_^2^ − 0.1X_3_^2^ (5)

**Figure 2:**
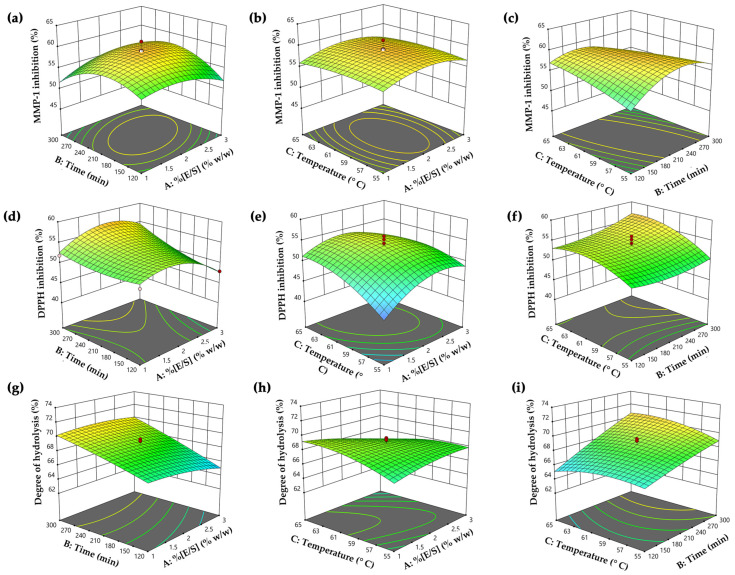
The three-dimensional surfaces and contour plots of independent hydrolysis variables, including enzyme–substrate concentration (E/S) (X_1_), hydrolysis time (X_2_), and temperature (X_3_) on the responses, including (**a**–**c**) Y_1_: matrix metalloproteinase (MMP)-1 inhibitory activity, (**d**–**f**) Y_2_: 2,2-diphenyl-1-picrylhydrazyl radical (DPPH^●^) scavenging activity, and (**g**–**i**) Y_3_: degree of hydrolysis.

The quadratic regression equation demonstrated a robust fit, as evidenced by the R^2^ and adjusted R^2^ values of 0.95 and 0.91, respectively. This value indicated that 91% of the variability in the data could be described by the model, with minimal influence from experimental error. The lack of fit value was statistically insignificant (*p* > 0.05), confirming the equation’s reliability. The most significant effect of PH on MMP-1 inhibitory activity was the quadratic term of time (X_2_^2^) (*p* < 0.0001), as well as the interactions between enzyme–substrate concentration and hydrolysis time (X_1_X_2_), the interaction between hydrolysis time and temperature (X_2_X_3_), and quadratic terms of enzyme–substrate concentration (X_1_^2^), which were also statistically significant (*p* < 0.05). The analysis employed three-dimensional response surfaces and contour plots to elucidate the impact of variables on MMP-1 inhibitory activity, as shown in Figure 2a–c. The visuals illustrated that MMP-1 inhibitory activity initially rose with higher enzyme–substrate concentration, hydrolysis time, and temperature, reaching a peak before declining. Notably, prolonged hydrolysis time and higher temperatures were associated with decreased anti-MMP-1 activity (Figure 2c). This decline could be attributed to excessive hydrolysis time and temperature, which caused the degradation of bioactive peptides into inactive peptides or amino acids, resulting in the loss of this inhibitory activity [51]. Therefore, the optimal temperature and time for hydrolysis were crucial for generating peptides with high MMP-1 inhibitory activity. This finding was in alignment with numerous previous studies that utilized the Alcalase^®^ enzyme, either independently or in combination with other proteinase enzymes, under optimal conditions to produce PH, demonstrating notable MMP-1 inhibitory activity [52,53,54].

#### 3.1.2. DPPH^●^ Scavenging Activity

The DPPH^●^ inhibitory capabilities of PH displayed values ranging from (42.2 to 59.3)% under diverse hydrolysis conditions and demonstrated a quadratic relationship with hydrolysis variables. This relationship was supported by a highly significant model (*p* < 0.0001). Through multiple regression analysis, a second-order polynomial equation in coded form was expressed as Equation (6), representing the relationship between DPPH^●^ scavenging activity and various variables.

Y_2_ = 53.8 + 1.0X_1_ + 1.0X_2_ + 2.3X_3_ + 1.5X_1_X_2_ − 1.6X_1_X_3_ + 0.1X_2_X_3_ − 3.3X_1_^2^ + 1.0X_2_^2^ − 2.1X_3_^2^ (6)

The quadratic regression equation exhibited a strong fit, with R^2^ and adjusted R^2^ values of 0.94 and 0.88, respectively. The lack of fit value was statistically insignificant (*p* > 0.05), affirming the reliability of the equation. This finding also showed that a quadratic term of enzyme–substrate concentration (X_1_^2^) was the most significantly influencing anti-DPPH^●^ activity (*p* < 0.0001). Additionally, statistically significant impacts were observed in all variables (*p* < 0.05), except for the interaction between time and temperature (X_2_X_3_). The influence of hydrolysis variables on DPPH^●^ scavenging was illustrated through three-dimensional surfaces and contour plots in Figure 2d–f. The plots revealed no significant differences in DPPH^●^ inhibitory activity at low enzyme–substrate concentrations (E/S 1% *w*/*w*). However, increased activity was observed with a higher enzyme–substrate concentration and a longer hydrolysis time (Figure 2d). Moreover, reduced DPPH^●^ inhibitory activity was noted with excessive enzyme–substrate concentration, possibly due to the limitation of protein cleavage caused by enzyme saturation [55]. Furthermore, Figure 2f illustrates that prolonged hydrolysis time and higher temperature led to increased DPPH^●^ activity, likely due to more extensive hydrolysis reactions and the release of bioactive peptides, contributing to higher antioxidative activity [56]. Our finding was supported by the previous research from Hall et al. (2018), indicating that cricket hydrolysates (*Gryllodes sigillatus*) prepared with Alcalase^®^ exhibited higher DPPH^●^ scavenging activity when the enzyme–substrate concentrations were in the range from (0.25 to 0.5)%, and lower activity at the excess concentration (3%) [27]. This observation suggested that the superior scavenging activity was linked to the use of an optimal enzyme–substrate concentration in the hydrolysis reaction. Therefore, this study highlighted the critical role of hydrolysis parameters for the antioxidant potential of PH production.

#### 3.1.3. Degree of Hydrolysis

The degree of hydrolysis results of PH, ranging from (63.6 to 72.3)%, showed a quadratic relationship with hydrolysis variables under diverse hydrolysis conditions. This pattern was strongly supported by a highly significant model (*p* < 0.05). The correlation between the degree of hydrolysis and various variables is expressed in Equation (7) through a second-order polynomial equation in coded form:

Y_3_ = 68.6 − 0.5X_1_ + 2.1X_2_ + 0.2X_3_ + 0.4X_1_X_2_ − 1.0X_1_X_3_ + 0.7X_2_X_3_ − 0.4X_1_^2^ − 0.2X_2_^2^ − 0.7X_3_^2^ (7)

The variability of the quadratic relationship between degree of hydrolysis and hydrolysis variables was confirmed by an R² of 0.92 and an adjusted R² of 0.85. The lack of fit value was statistically insignificant (*p* > 0.05), affirming the reliability of the equation. This result indicated that the model effectively explained approximately 85% of the variability in the degree of hydrolysis, with minimal influence from experimental error. Hydrolysis time (X_2_) was identified as the most significant factor *(p* < 0.0001) influencing the degree of hydrolysis value. Additionally, the interaction effects of enzyme–substrate concentration and temperature (X_1_X_3_), the interaction of time and temperature (X_2_X_3_), and the quadratic term of temperature (X_3_^2^) were also found to be significant contributors to the degree of hydrolysis (*p* < 0.05). The impact of variables on the degree of hydrolysis was illustrated using three-dimensional response surfaces and contour plots, as shown in Figure 2g–i. The finding indicated that higher hydrolysis time contributed to an increased degree of hydrolysis values, especially when the enzyme–substrate concentration was below 2.5% (Figure 2g). Beyond this point, a plateau in the degree of hydrolysis value was observed, suggesting a steady rate, likely due to substrate depletion [57]. The hydrolytic reaction was contingent on the availability of susceptible peptide bonds, with the enzymatic activity focused on breaking down the physical structure of the protein molecules [58]. As a result, in the early phases of hydrolysis, numerous peptide linkages were suddenly broken, resulting in a higher hydrolysis degree. This process tended to slow down as the available peptide bonds became depleted. Figure 2i illustrates that extended hydrolysis time and elevated temperatures were correlated with higher hydrolysis degree, indicating increased production of smaller peptides with enhanced antioxidant activity. The observed degree of hydrolysis values in our study were greater than those reported in a previous investigation, where the PH showed a degree of hydrolysis ranging from (32.2 to 57.9)% under various hydrolysis conditions [59]. In another study, the degree of hydrolysis values was determined to be 46% and 33% in cricket protein hydrolysate prepared by Proteinase A and Flavouzyme^®^ after a 2 h hydrolysis period [60]. Moreover, another study by Calzada Luna reported degree of hydrolysis values ranging from (8.11 to 13.98)% in *A. domesticus* hydrolysates prepared with Alcalase^®^ [61]. Variations in parameters such as enzyme-to-substrate ratio (E/S), temperature, pH, reaction duration, and the method used for measuring the degree of hydrolysis values might contribute to the observed differences in the degree of hydrolysis values [62].

### 3.2. Predictive Optimal Hydrolysis Conditions and Verification

The optimization of hydrolysis conditions and the prediction of responses were conducted using Design Expert 13.0 software. Achieving optimal conditions through this optimization process is crucial for producing bioactive peptides with the desired properties [63]. The optimal conditions for producing PH with the highest MMP-1 and DPPH^●^ inhibitory activities was determined to be an enzyme–substrate concentration of 2.1% *w*/*w*, a temperature of 61.5 °C, and a duration of 227 min at a pH of 7.5. To validate the model, actual experiments were conducted under the predicted optimal conditions. The predicted and experimental values are presented in Table 3. The results demonstrated that the experimental value agreed with the predicted value within a 95% confidence interval. This alignment signified the successful application of RSM in optimizing the hydrolysis conditions of PH. Therefore, the model was considered reliable and had the effective potential to predict PH production with robust anti-skin aging activity.

### 3.3. Chemical Composition of CE, PC, and PH

#### 3.3.1. Total Protein Content and MW Distribution

To generate PH with anti-skin aging properties, proteins were extracted and concentrated from *A. domesticus*, resulting in the formation of CE and PC with total protein contents of 60.9 ± 1.7% and 73.0 ± 0.3% *w*/*w* on a dry basis, respectively. Subsequently, PC was hydrolyzed with Alcalase^®^ under the optimized hydrolysis conditions, yielding PH with a total protein content of 55.5 ± 0.9% *w*/*w* on a dry basis, which was lower than CE and PC. The observed results could be attributed to the underlying principle of the protein determination method, the BCA assay, which relies on the interaction of peptide bonds with Cu^2+^ and bicinchoninic acid [64]. Enzymatic hydrolysis might cleave peptide bonds within the intact protein, thereby diminishing the reaction involving Cu^2+^ and bicinchoninic acid. The results aligned with a previous investigation, indicating protein concentrations of 61.3% and 71.7% in defatted flour and protein isolate from *A. domesticus*, respectively. Furthermore, PH from *A. domesticus*, produced with the Alcalase^®^ enzyme, exhibited a protein content of 57.97 mg/mL [65].

**Figure 3:**
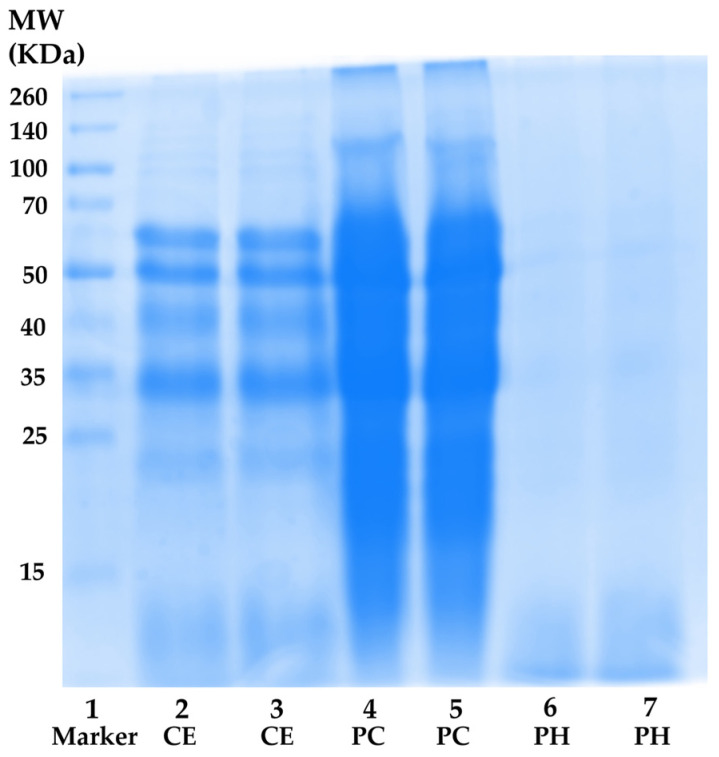
Protein molecular weight distributions. Lane 1: protein molecular weight standard; lanes 2 and 3: *A. domesticus* crude extract (CE); lanes 4 and 5: *A. domesticus* protein concentrate (PC); and lanes 6 and 7: *A. domesticus* protein hydrolysate (PH).

Nevertheless, the total protein content result aligned with the MW distribution profile obtained through SDS-PAGE electrophoresis, as shown in Figure 3. CE (lanes 2 and 3) exhibited a spectrum of proteins with diverse MWs ranging from (10 to 260) kDa. The most prominent protein bands in CE appeared at approximately MW of 60 kDa, 50 kDa, 40 kDa, and 33 kDa, suggesting the presence of β-glycosidases (60 kDa), tubulins (50 kDa), actin (42 kDa), monomeric arginine kinase (41 kDa), and tropomyosin (33 kDa), respectively [66,67,68]. In the case of PC (lanes 4 and 5), more intense protein bands were observed compared to CE at the same concentration. This outcome could be attributed to the higher protein content in PC, indicating the successful concentration of proteins in CE. Regarding PH (lanes 6 and 7), the protein bands nearly vanished, giving way to a diffuse band with MW below 15 kDa. The result was consistent with the previous report, which revealed that multiple protein bands with MWs ranging from (212 to 14.4) kDa were observed in unhydrolyzed cricket (*Gryllodes sigillatus*) protein, while dominant bands with MW below 14.4 kDa were detected in PH [27]. Furthermore, the protein pattern was similarly observed in black crickets (*Gryllus assimilis*), with a diverse range of protein MWs found in both protein concentrate and non-hydrolyzed protein. Additionally, the predominant composition of the PH consisted of polypeptide chains with MW under 14 kDa, accompanied by a reduction in the intensity of high-molecular-weight protein bands [37]. This finding confirmed that the hydrolysis process successfully broke down native protein under optimal hydrolysis conditions into short fragment peptides and amino acids, which possessed beneficial skin aging inhibitory activities.

#### 3.3.2. Amino Acid Composition

The amino acid composition of PH is detailed in Table 4. Seventeen amino acids were detected in the PH. However, tryptophan identification was hindered due to its destruction by acid hydrolysis under the analysis conditions. The most abundant amino acids in PH were glutamic acid (Glu) and its derivative glutamine (Gln), followed by aspartic acid (Asp) and its derivatives asparagine (Asn), leucine (Leu), alanine (Ala), and serine (Ser), respectively. Moreover, PH comprised 36.7% of hydrophobic amino acids, such as alanine (Ala), valine (Val), isoleucine (Ile), leucine (Lue), phenylalanine (Phe), proline (Pro), and methionine (Met). These hydrophobic amino acids play a crucial role in the antioxidant properties of PH through diverse mechanisms, incorporating proton donation, electron donation, and inhibitory abilities against lipid peroxidation [69]. Furthermore, aromatic amino acids, such as tyrosine (Tyr) and tryptophan, possess inherent abilities to scavenge free radicals [70]. Indeed, the MMP-1 inhibitory activities observed in PH could be attributed to specific amino acids. Pro is known for its potential to maintain the secondary structure of the polypeptide chain, thereby influencing the overall stability of collagen [71]. The amino acid composition of PH in this study showed variations compared to Grossman’s study (2021), where Leu, Val, Lys, and Glu were identified as the most abundant amino acids in PH, respectively [60]. Such discrepancies could arise from the use of different enzyme types for hydrolysis. Additionally, the amino acid composition in PH was influenced by various factors such as the initial protein substrate, the hydrolysis conditions applied, and the method employed for amino acid determination [72,73,74].

### 3.4. Anti-Skin Aging Activity

In this study, CE, PC, and PH were investigated for anti-skin aging activity, comprising MMP-1 inhibitory, hyaluronidase inhibitory, DPPH^●^ scavenging, and lipid peroxidation inhibitory effects, and the results were expressed as IC_50_ values for each activity, as presented in Table 5. Among the samples, PH exhibited the significantly highest activities against MMP-1, hyaluronidase, DPPH^●^, and lipid peroxidation (*p* < 0.05), with IC_50_ values of 15.5 ± 1.8, 36.7 ± 3.5, 91.0 ± 6.2, and 121.6 ± 7.6 µg/mL, respectively. Despite having higher IC_50_ values than the positive control, except for anti-hyaluronidase activity, a consistent pattern emerged where the IC_50_ of PH was the significantly lowest (*p* < 0.05), followed by PC and CE in all activities, respectively. This study suggested that proteins were the major compounds responsible for the activity, and enzymatic hydrolysis could enhance efficacy by releasing bioactive peptides during the hydrolysis. This result was consistent with the study by Quinteros et al. (2022), which highlighted that the antioxidant activity of protein concentrate derived from crickets surpassed that from cricket flour [38]. A study by Hall et al. (2018) also found a significant positive impact of antioxidant activities on cricket protein hydrolyzed by the Alcalase^®^ enzyme compared to unhydrolyzed protein. The study reported that the hydrolysis process could enhance antioxidant activity in the DPPH, ABTS, and FRAP assays, with values of 1490, 663, and 991 μmol TE/mg, respectively [27]. Moreover, the previous study supported the idea that cricket hydrolysate has significant efficacy in inhibiting lipid oxidation in the goat emulsion samples during storage on days 7 and 14, which exhibited significantly lower values for thiobarbituric acid reactive substances and free fatty acids compared with the control (*p* < 0.05) [75].

In the context of enzymatic inhibitory activity related to skin aging, our finding aligned with prior research indicating that peptides identified from macroalgae PH, specifically Asn-Arg-Asp-Tyr and Arg-Asp-Arg-Phe, exhibited concentration-dependent inhibition of collagenase and the IC_50_ values for these peptides were reported as 0.95 mM and 0.84 mM, respectively [54]. This finding was reinforced by Kim (2014), who noted that low-molecular-weight peptides from horse leg bone hydrolysates (at 100 mg/mL) exhibited collagenase inhibitory activity, achieving a 91.32% inhibition rate [76]. Regarding hyaluronidase inhibitory activity, PH exhibited activity comparable to oleanolic acid, a well-known hyaluronidase inhibitor. This result corresponded with a study by Wang et al. (2020), who reported that hyaluronidase inhibitory activity, ranging from (16 to 50)%, was exhibited by small-molecular-weight peptides derived from salmon by-products and this activity was influenced by the type of enzyme used in the hydrolysis process [77]. Chen et al. (2020) revealed that rice PH, created by bacterial amylase and protease, could inhibit the hyaluronidase enzyme. This inhibition occurred through competitive binding, where the hydrolysate competed with hyaluronic acids to bind to the enzyme effectively [78]. Thus, the enzymatic treatment has proven to be an efficient process for enhancing the skin aging inhibitory properties of *A. domesticus* protein, suggesting its potential as an optional natural bioactive ingredient for novel cosmeceuticals.

### 3.5. Anti-Inflammatory Activity

The IKK/NF-κB signaling pathway plays a pivotal role as a key mediator in the aging process, responding to a variety of factors, including genotoxic, oxidative, and inflammatory elements [79]. This pathway is responsible for regulating the expression of cytokines associated with skin aging, such as MMP-1, MMP-3, MMP-9, and tumor necrosis factor-alpha. The disruption of the delicate balance in the regulation of these cytokines results in the degradation of the ECM, contributing to the acceleration of skin aging [80]. Consequently, the inhibition of NF-κB has been explicitly linked to a delayed onset of physiological and pathological manifestations of skin aging [81].

**Figure 4:**
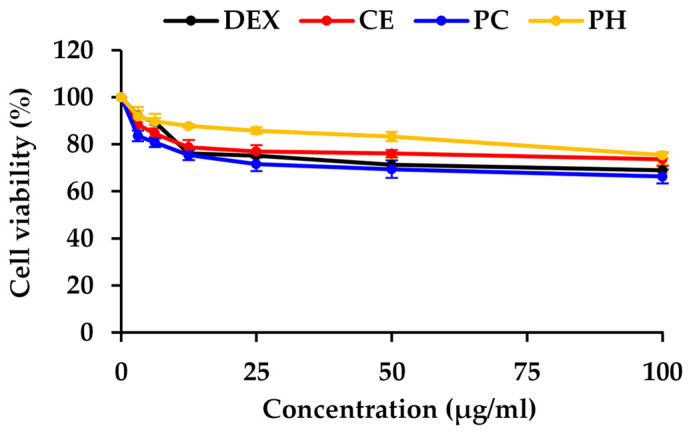
The dose–response curve of dexamethasone (Dex), *A. domesticus* crude extract (CE), *A. domesticus* protein concentrate (PC), and *A. domesticus* protein hydrolysate (PH) on the viability of HaCaT cell lines. The data are shown as mean ± SD (n = 3).

In this study, dexamethasone (Dex), CE, PC, and PH were assessed for cell viability in HaCaT cells, chosen as representative human skin cells, and the result is depicted in the dose–response curve in Figure 4. Dexamethasone, a synthetic glucocorticoid, served as the positive control in the study due to its well-established ability to inhibit the expression of the NF-κB protein. Based on non-cytotoxic doses, the concentrations for assessing NF-κB inhibitory activity were selected from the concentrations reducing cell viability by 20% (IC_20_ values), with 11 ± 1, 14.2 ± 6.5, 7.2 ± 2.3, and 69.8 ± 6.0 μg/mL for Dex, CE, PC, and PH, respectively. The previous study also observed a similar trend that unhydrolyzed protein from black crickets (0.5 mg/mL) exhibited lower cell viability in RAW 264.7 cells compared to the PH [82]. This observation strongly implied that enzymatic hydrolysis may contribute to an improved safety profile of the cricket protein.

**Figure 5:**
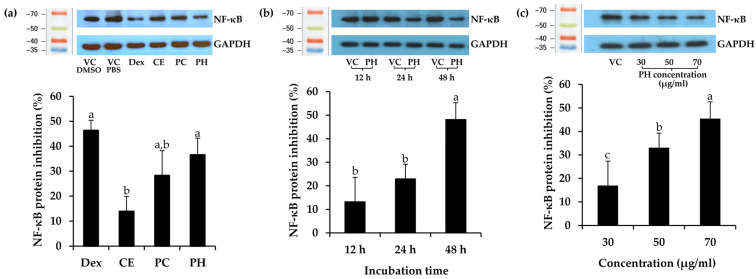
Effect of dexamethasone (Dex), *A. domesticus* crude extract (CE), *A. domesticus* protein concentrate (PC), and *A. domesticus* protein hydrolysate (PH) on nuclear factor-kappaB (NF-κB) protein expression in HaCaT cells compared with vehicle control (VC). (**a**) The levels of NF-κB protein following treatments with Dex, CE, PC, and PH at the concentration reduced cell viability by 20% (IC_20_ values) for 48 h. (**b**) The levels of NF-κB protein following treatments with PH at 70 μg/mL for 12, 24, and 48 h. (**c**) The levels of NF-κB protein following treatments with PH at 30, 50, and 70 μg/mL for 48 h. The levels of NF-κB protein were normalized using GAPDH protein levels. The data are shown as mean ± SD (n = 3). The lowercase letters (a, b, and c) indicate significant variability between the samples. Statistical significance was analyzed using a one-way ANOVA followed by a post hoc Tukey test (*p* < 0.05).

Subsequently, Dex, CE, PC, and PH at the concentration of IC_20_ values were determined for NF-κB inhibitory activity and are depicted in Figure 5a. Remarkably, PH demonstrated the highest NF-κB suppression activity among the others, inhibiting NF-κB by 36.5 ± 6.7% compared to the vehicle control PBS. This effect was notably comparable to Dex, a widely recognized anti-inflammatory, exhibiting an inhibition of 46.3 ± 4.1% compared to the vehicle control DMSO. Furthermore, the trend of NF-κB suppression activity was observed in PC and CE, with inhibitions of 25.8 ± 1.8% and 13.6 ± 5.5%, respectively. These results strongly indicated that the protein derived from *A. domesticus* was the primary compound responsible for NF-κB suppression, and enzymatic hydrolysis served to enhance its anti-inflammatory activity. Therefore, PH was chosen for a time-dependent investigation into NF-κB inhibition, as shown in Figure 5b. The results showed that the inhibition of NF-κB protein levels after PH treatment for 12, 24, and 48 h was 13.2 ± 10.4%, 23.0 ± 6.2%, and 48.2 ± 7.1%, respectively. The result emphasized that the duration of incubation significantly influenced NF-κB suppression (*p* < 0.05), exhibiting a time-dependent effect, with the most pronounced effect observed at 48 h. Consequently, further exploration of concentration effects at the time of 48 h was performed. Various concentrations of PH were investigated for NF-κB inhibition, as shown in Figure 5c. PH exhibited NF-κB protein suppression levels of 17.0 ± 5.6%, 33.1 ± 4.2%, and 45.5 ± 2.9% at concentrations of 30, 50, and 70 μg/mL, respectively. Therefore, PH exhibited concentration-dependent anti-NF-κB activity, with the most effective concentration observed at 70 µg/mL. The present finding was consistent with an earlier study indicating that cells pre-treated with cationic peptides from cricket PH exhibited reduced levels of NF-κB in RAW 264.7 macrophage cells compared to the control group [83]. The findings from Zielińska et al. (2017) further support these observations, revealing that the fraction derived from *G. sigillatus* protein preparation, which displayed the highest antiradical activity, also served as the most effective inhibitor of cytokine-related inflammation, including lipoxygenase (IC_50_ value of 0.13 µg/mL) and cyclooxygenase-2 (IC_50_ value of 0.26 µg/mL) [84]. As a result, the anti-inflammatory potential of the *A. domesticus* protein could be elevated through enzymatic hydrolysis, exerting a direct influence on the NF-κB inhibition pathway intricately connected to the skin aging process.

The findings from this study revealed the remarkable efficacy of PH in attenuating NF-κB protein levels, aligning with its observed inhibition of MMP-1 activity. However, the regulation of MMP-1 involved not only the NF-κB pathway but also the mitogen-activated protein kinase (MAPK) family, MAPK/activator protein 1 (AP-1), transforming growth factor/suppressor of mothers against decapentaplegic (TGF/Smad) pathways, etc. [85,86,87]. Hence, the strategic targeting of MAPK signaling pathways would be suggested for further study to present a promising avenue for inhibiting MMP-1 expression, thereby addressing skin aging and inflammation.

### 3.6. Anti-Skin Aging Bioactive Peptides Derived from PH

#### 3.6.1. Anti-Skin Aging Fractions from PH

PH was fractionated into five fractions using an ultrafiltration technique with membrane MW cutoffs of 1, 3, and 10 kDa, resulting in PH-I (MW < 1 kDa), PH-II (MW = (1 to 3) kDa), PH-III (MW = (3 to 10) kDa), and PH-IV (MW > 10 kDa), respectively. Each fraction was assessed for anti-skin aging properties via MMP-1 inhibitory and DPPH^●^ scavenging activities. While these assays alone may not fully capture the complexity of anti-aging mechanisms, they served as practical tools to identify the bioactive fraction and establish a preliminary understanding of its potential. Notably, among these fractions, PH-I demonstrated the significantly highest MMP-1 inhibitory and DPPH^●^ scavenging activities (*p* < 0.05), with the values of 6.2 ± 0.1 µg OAE/mg protein and 3.5 ± 0.1 µg AAE/mg protein, respectively (Table 6). The obtained results, aligned with the previous finding of Hong et al. (2019), indicated that the peptide fraction with the lowest MW (< 3 kDa) from porcine by-products exhibited enhanced collagenase inhibition compared to the unfractionated hydrolysate [88]. They also found that collagen peptides produced with Alcalase^®^ showed significantly greater anti-collagenase activity in the fraction with a MW below 3 kDa (61.9%) compared to the higher MW fraction (54.4%) (*p* < 0.05). Similarly, a consistent trend in antioxidant activity was observed. Fashakin’s study showed that the smallest MW fraction (<3 kDa) of cricket protein hydrolyzed with Alcalase^®^ demonstrated the highest anti-radical ABTS activity with the values of 0.45 µmol Trolox eq./g, surpassing the activity of fractions with MW of (3 to 10) kDa and >10 kDa [89]. This consistency with prior research highlighted the trend that lower MW peptides often possess higher bioactivity, likely due to the presence of smaller peptide sequences facilitating binding to target proteins or free radicals [90]. Hence, this study suggested that the primary contributors to the anti-skin aging activity in PH were the low-molecular-weight peptides, especially those below 1 kDa. Consequently, further isolation of PH-I was carried out through gel filtration chromatography.

#### 3.6.2. Anti-Skin Aging Peptides Isolated from PH-I Fraction

**Figure 6:**
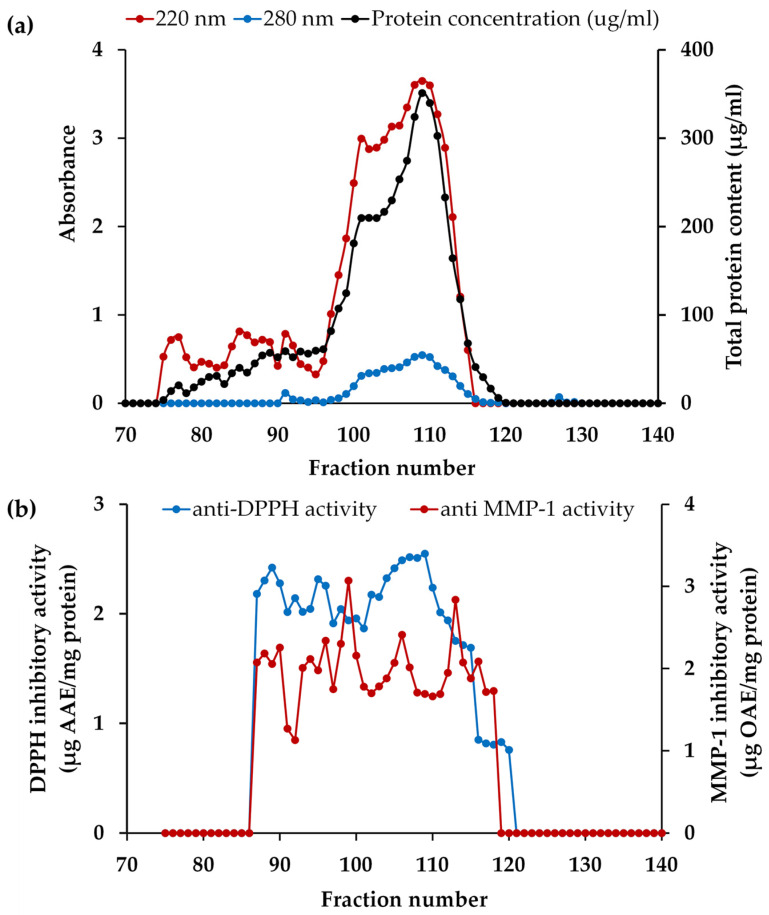
The chromatogram of the fractions from PH-I isolated from Sephadex G-25: (**a**) absorbance values of 220 nm and 280 nm and protein concentration of each fraction; (**b**) matrix metalloproteinase (MMP)-1 inhibitory and 2,2-diphenyl-1-picrylhydrazyl radical (DPPH^●^) scavenging activities of each fraction.

Prior to PH isolation, blue dextran (2,000,000 Da), insulin B (3495.9 Da), vitamin B12 (1355.5 Da), glycine-tyrosine (238.2 Da), and tyrosine (181 Da) were employed as standard compounds for affirming the correlation between elution volume and their respective MWs on a Sephadex G-25 column. According to the principles of size exclusion chromatography, larger protein molecules were excluded from the internal volume and eluted first from the column, while smaller protein molecules, with the ability to access the internal volume, were eluted later [91]. Consequently, the elution volumes were 119 mL for blue dextran, 195 mL for insulin B, 220 mL for vitamin B12, 274 mL for glycine-tyrosine, and 302 mL for tyrosine, respectively. Then PH-I was subjected to isolation through gel filtration chromatography and the absorbances at 220 nm and 280 nm, representing peptide bonds and aromatic amino acids in peptides, respectively, were monitored for each fraction, as illustrated in Figure 6a. The initial fraction was devoid of detectable compounds until fraction number 75, as evidenced by the A220 nm results, which indicated the presence of peptide bonds in fraction numbers 75 to 115. This range corresponded to approximately MW between (185 and 4920) Da, as compared to the chromatogram of the protein MW standard. Both the A220 and A280 results exhibited a similar pattern, but the A280 results consistently showed lower values. The results of protein concentrations were consistently mirrored with A220 nm results, confirming that the A220 nm results indeed represent the presence of peptide bonds in the proteins or peptides. Numerous researchers have demonstrated that Alcalase^®^ was a highly effective enzyme in the preparation of PH, yielding peptides with a broad range of MWs and diverse biological activities [92,93].

Following isolation, each fraction was determined for the MMP-1 inhibitory and DPPH^●^ scavenging activities, and the results are depicted in Figure 6b. Remarkably, the most significant collagenase inhibitory activity was detected in fractions 99 (estimated MW = 853 Da) and 113 (estimated MW = 307 Da), with MMP-1 inhibitory activities of 3.1 ± 0.2 and 2.8 ± 0.1 µg OAE/mg protein, respectively (*p* < 0.05). Furthermore, fraction 106 (estimated MW = 512 Da) proved to be particularly noteworthy, as it demonstrated its dual functionality regarding high DPPH^●^ scavenging and moderate anti-MMP-1 capabilities with the values of 2.5 ± 0.1 µg AAE/mg protein and 2.4 ± 0.1 µg OAE/mg protein, respectively. This result corresponded with the previous study reported that low MW (<1 kDa) and intermediate MW ((1 to 10) kDa) fractions of peptides from poultry feathers significantly decreased the expression levels of MMP-1 and MMP-13 caused by UVB radiation in a dose-dependent manner in human dermal fibroblasts [94]. Jin et al. (2018) also found that low-molecular-weight peptides (<1 kDa), obtained from the keratin peptide fraction possessed activity against matrix metalloproteinases (MMPs) [95]. Therefore, the peptide fractions 99, 106, and 113 were chosen for peptide identification. Although solely relying on MMP-1 inhibitory and DPPH^●^ scavenging activities may not provide a comprehensive assessment of the anti-aging properties of a specific material, skin aging involves a multitude of complex mechanisms. Both assays served as important tools for characterizing the bioactive fractions, laying the foundation for further investigations into their broader anti-aging mechanisms.

#### 3.6.3. Amino Acid Sequences of Anti-Skin Aging Peptides from PH

A comprehensive analysis identified a total of 95 peptide sequences from fractions 99, 106, and 113. Meeting the criteria of an ALC score ≥ 95, seventeen specific sequence peptides were observed, as outlined in Table 7. These peptides exhibited a MW range between 703 and 1562 Da, with lengths varying from 7 to 16 amino acids. This result was consistent with a prior study indicating that most bioactive peptides tend to have lengths ranging from 2 (dipeptides) to 20 amino acid residues [96]. Additionally, these bioactive peptides typically possess a molecular mass falling within the range of (0.4 to 2) kDa [97]. Since ALC represents the mean value of the local confidence score at each amino acid position, higher ALC scores indicate greater overall confidence in the de novo sequence for a given spectrum [98]. Emphasizing peptide sequences with the highest ALC scores for each fraction, three particularly potent anti-skin aging peptides were identified: Ala-Val-Thr-Lys-Ala-Asp-Pro-Tyr-Thr-Asp-Gln (AVTKADPYTDQ: 1208 Da) in fraction 99, Thr-Val-Met-Glu-Leu-Asn-Asp-Leu-Val-Lys-Ala-Phe (TVMELNDLVKAF: 1379 Da) in fraction 106, and Val-Pro-Leu-Leu-Glu-Pro-Trp (VPLLGPW: 780 Da) in fraction 113.

The observed efficacy against skin aging in the selected peptide fractions was not solely a result of their peptide size or MW. This effectiveness was attributed to the diverse amino acids and specific peptide sequences. However, our understanding of the molecular mechanisms underlying the impact of peptides on anti-skin aging activity is currently limited, and various theories have been proposed [99]. In a prior study, WNLNP identified peptides from oysters exhibiting potent anti-photoaging effects on UVB-irradiated HaCaT cells [100]. Its protective mechanism involved inhibiting reactive oxygen species production, reducing MMP-1 expression, and enhancing pro-collagen I content production. Molecular docking analysis revealed WNLNP’s interaction with NF-κB (p65) and MMP-1, forming five and seven hydrogen bonds, respectively. Aguilar-Toalá and Liceaga (2020) identified specific sequences, APHWYTN, DQNPRSF, GDAHWAY, GDAHWTY, GDAHWVY, GFEWITF, and KKLKRVYV, in chia seed peptides that demonstrated inhibitory effects on skin-aging enzymes, including elastase, tyrosinase, hyaluronidase, and collagenase [101]. These peptides showed mixed-type inhibition against elastase and hyaluronidase and a noncompetitive pattern against collagenase and tyrosinase. They suggested their capability to interact with active sites or other regions of these enzymes, preventing substrate binding. Additionally, chelating agents possessed the capability to inhibit metalloproteinase enzymes such as collagenase [102], given that sorghum grain peptides demonstrated chelating capacity, which potentially inhibited collagenase by chelating zinc ions in its active site [103]. Several studies employed cell lines to explore the mechanism of action of anti-aging peptides. The peptides identified from silver carp skin, such as GPPGPPGTPGPQ, SGLPGPIGPPGPR, and GLPGPIGPPGPR, demonstrated anti-photoaging activity by exhibiting significant DPPH^●^ and hydroxyl radical scavenging. Moreover, these peptides could inhibit MMP-1 secretion in mouse fibroblast cell lines [104]. In additional studies, peptides obtained from Pacific cod skin gelatin hydrolysate were found to inhibit MMP-1, phosphorylated-extracellular signal-regulated kinase, and phosphorylated-p38 MAPK in UVB-induced mouse skin fibroblasts using enzyme-linked immunosorbent assays [105].

One of the most promising activities of bioactive peptides is their antioxidant function. The effectiveness of these peptides in antioxidant role was associated with their structural characteristics, which involved factors like MW, amino acid sequence, and hydrophobicity [106]. Notably, this study revealed that three identified anti-skin aging peptides coexisted with hydrophobic amino acids, including Val, Ala, Leu, and Pro, in their sequences. Additionally, aromatic amino acids were predominantly located at the terminus of the peptide chains. This result aligned with the previous study of Li et al. (2011), which reported that the N-terminus of antioxidant peptides typically consisted of hydrophobic amino acids or tyrosine [107]. This characteristic enabled these peptides to effectively scavenge protons and radicals. Another study showed that the composition and proportion of hydrophobic amino acids in the peptide sequence were related to the antioxidant activity, with stronger antioxidant activity [108]. However, few studies have revealed the amino acid sequence with antioxidant capabilities in the PH derived from crickets. Fashakin et al. (2023) revealed that ten identified peptides in PH from black cricket (*Gryllus bimaculatus*) were TEAPLNPK, EVGA, KLL, TGNLPGAAHPLLL, AHLLT, LSPLYE, AGVL, VAAV, VAGL, and QLL [89]. The amino acid composition showed a predominance of hydrophobic amino acids ((50 to 100)%), contributing to the peptides’ significant antioxidant activity. Moreover, a total of 28 peptides were identified in cricket PH, featuring sequences like VGPPQ, YKPRP, and PHGAP. Most of these identified sequences contained tripeptides (GAP and GPP), showcasing multifunctional activities such as antioxidant, anti-hypertensive, antimicrobial, anti-glycemic, and immunomodulatory properties [83]. Furthermore, the study of de Matos et al. (2022) also identified AGDDAPR and WDDMEK in the PH of *Gryllus assimilis*, demonstrating antioxidant, antidiabetic, and antihypertensive activities [37]. Compared to other research, variations in bioactive peptide sequences and length were influenced by the specificity of enzymes when cleaving the protein under various hydrolysis conditions. This process led to the emergence of distinct bioactivities in bioactive peptides [30]. Additionally, the characteristics and biological activities of bioactive peptides were significantly influenced by several factors, such as the protein source, hydrolysis conditions, hydrolysis technique, and the type of enzyme used [109]. Considering these factors, the exploration of new protein sources with positive effects becomes essential. Therefore, *A. domesticus* emerges as a promising source of PH with anti-skin aging properties, displaying potential applications in the cosmeceutical industry.

## 4. Conclusions

In this study, PH was generated by Alcalase^®^-mediated hydrolysis through optimizing conditions using an RSM approach. The most promising PH, with the significantly highest degree of hydrolysis as well as MMP-1 and DPPH^●^ inhibition, was achieved at optimal conditions: an E/S of 2.1% (*w*/*w*), a temperature of 61.5 °C, and a duration of 227 min. Within PH, abundant amino acids, including Glu, Asp, and Leu, were identified. The MW distribution of PH was demonstrated to be below 15 kDa, confirming the success of the hydrolysis process. Notably, PH exhibited the significantly greatest biological activities related to the anti-skin aging properties, comprising inhibitory activities against MMP-1 (IC_50_ = 15.5 ± 1.8 µg/mL), hyaluronidase (IC_50_ = 36.7 ± 3.5 µg/mL), DPPH radical (IC_50_ = 91.0 ± 6.2 µg/mL), and lipid peroxidation (IC_50_ = 121.6 ± 7.6 µg/mL). Furthermore, PH displayed the significantly most potent anti-inflammatory activity through inhibiting the NF-κB protein in time- and dose-dependent manners. Following isolation and identification, three promising anti-skin aging peptides, including AVTKADPYTDQ, TVMELNDLVKAF, and VPLLGPW, were identified and found to exhibit notable MMP-1 inhibitory and DPPH^●^ scavenging activities. These findings suggested that PH from *A. domesticus*, produced with the Alcalase^®^ enzyme under optimal conditions, holds potential as a valuable source of bioactive compounds for the cosmeceutical industry. This study marks a significant departure from the traditional use of insects, extending their applications beyond the food industry to the cosmeceutical area. However, to thoroughly understand the potential of the novel anti-skin aging peptides identified in this research, additional investigations into their mechanisms are essential. Furthermore, conducting clinical trials to assess their safety and effectiveness in skincare is imperative.

## Figures and Tables

**Table 1:** The actual values of independent variables employed for optimizing the hydrolysis conditions using Alcalase^®^ enzyme.

Run	Variable Factors		
	X_1_ (E/S: % *w*/*w*)	X_2_ (Time: min)	X_3_ (Temp: °C)
1	1.0	300.0	55.0
2	1.0	300.0	65.0
3	3.0	300.0	55.0
4	2.0	58.6	60.0
5	2.0	210.0	60.0
6	2.0	210.0	68.4
7	2.0	210.0	60.0
8	2.0	210.0	60.0
9	2.0	210.0	51.6
10	2.0	210.0	60.0
11	3.0	120.0	55.0
12	3.7	320.0	60.0
13	1.0	120.0	65.0
14	3.0	120.0	65.0
15	2.0	210.0	60.0
16	0.3	210.0	60.0
17	3.0	300.0	65.0
18	2.0	361.4	60.0
19	2.0	210.0	60.0
20	1.0	120.0	55.0

Note: X_1_ = enzyme–substrate concentration (E/S: % *w*/*w*), X_2_ = hydrolysis time (min), and X_3_ = temperature (°C).

**Table 2:** The experimental responses based on specified independent variables employed for optimizing the hydrolysis conditions using the Alcalase^®^ enzyme.

Run	Experimental Responses		
	Y_1_ (% MMP-1 Inhibition)	Y_2_ (% DPPH^●^ Inhibition)	Y_3_ (% DH)
1	53.6	42.2	66.9
2	48.3	51.9	71.2
3	58.2	51.6	69.1
4	48.2	54.6	63.8
5	61.3	55.3	69.5
6	57.1	53.2	66.0
7	57.8	52.7	68.2
8	59.1	51.9	68.3
9	55.8	43.9	66.9
10	58.6	56.1	69.2
11	50.0	48.1	66.2
12	52.5	46.7	66.1
13	55.8	50.8	67.3
14	54.0	48.0	63.6
15	59.0	52.0	69.2
16	53.3	43.6	68.7
17	52.9	53.0	69.4
18	58.0	59.7	72.3
19	59.0	54.3	67.5
20	52.4	46.7	65.6

Note: Y_1_ = matrix metalloproteinase (MMP)-1 inhibitory activity (%), Y_2_ = 2,2-diphenyl-1-picrylhydrazyl radical (DPPH^●^) scavenging activity (%), and Y_3_ = degree of hydrolysis (DH) (%). Each of the experiments was performed independently in triplicate under specified hydrolysis conditions and the data are shown as mean.

**Table 3:** The predicted values from Design Expert 13.0 software and the actual values from the experiment conducted under the optimized hydrolysis conditions for producing *A. domesticus* protein hydrolysate (PH).

	MMP-1 Inhibitory Activity (%)	DPPH^●^ Scavenging Activity (%)	DH (%)
Predicted values	58.8	54.5	68.9
Experimental values	59.3 ± 1.3	55.4 ± 2.6	65.7 ± 6.8

Note: MMP = matrix metalloproteinase, DPPH^●^ = 2,2-diphenyl-1-picrylhydrazyl radical, DH = degree of hydrolysis. Each of the experimental values was from an independent experiment performed in triplicate at optimal hydrolysis conditions. The data are shown as mean ± SD.

**Table 4:** The amino acid composition of *A. domesticus* protein hydrolysate (PH) prepared under the optimized hydrolysis conditions.

Amino Acid Composition of PH	Amount Based on a Dry Basis of PH (% *w*/*w*)
Essential amino acid	
Leucine	24.4
Valine	15.5
Lysine	14.7
Phenylalanine	10.7
Isoleucine	10.5
Threonine	8.7
Histidine	4.4
Methionine	3.7
Non-essential amino acid	
Glutamic acid and its derivative glutamine	41.0
Aspartic acid and its derivative asparagine	38.0
Alanine	17.8
Serine	17.4
Glycine	11.5
Arginine	11.4
Tyrosine	7.3
Proline	7.3
Cysteine	0.5
Total essential amino acids (%)	37.8
Total hydrophobic amino acids (%)	36.7

Note: PH = *A. domesticus* protein hydrolysate (PH).

**Table 5:** The IC_50_ of the anti-skin aging activity of *A. domesticus* crude extract (CE), *A. domesticus* protein concentrate (PC), *A. domesticus* protein hydrolysate (PH), oleanolic acid (OA), ascorbic acid (AA), and Trolox (TE).

Samples	IC_50_ (µg/mL)			
	MMP-1 Inhibition	Hyaluronidase Inhibition	DPPH^●^ Inhibition	Lipid Peroxidation Inhibition
CE	31.3 ± 0.8 ^a^	180.7 ± 2.0 ^a^	251.3 ± 6.2 ^a^	172.8 ± 2.2 ^a^
PC	19.2 ± 0.1 ^b^	89.8 ± 6.9 ^b^	143.7 ± 13.3 ^b^	157.6 ± 4.0 ^b^
PH	15.5 ± 1.8 ^c^	36.7 ± 3.5 ^c^	91.0 ± 6.2 ^c^	121.6 ± 7.6 ^c^
OA	2.7 ± 0.8 ^d^	28.7 ± 0.4 ^c^	-	-
AA	-	-	4.1 ± 0.0 ^d^	-
TE	-	-	-	4.8 ± 0.3 ^d^

Note: IC_50_ = half-maximal inhibitory concentration, MMP = matrix metalloproteinase, DPPH^●^ = 2,2-diphenyl-1-picrylhydrazyl radical, CE = *A. domesticus* crude extract, PC = *A. domesticus* protein concentrate, PH = *A. domesticus* protein hydrolysate, OA = oleanolic acid, AA = ascorbic acid, TE = Trolox. The data are shown as the mean ± SD (n = 3). The lowercase letters (a, b, c, and d) indicate significant variability among the samples. Statistical significance was analyzed using a one-way ANOVA followed by a post hoc Tukey test (*p* < 0.05).

**Table 6:** The MMP-1 inhibitory and DPPH^●^ scavenging activities of the fractions from *A. domesticus* protein hydrolysate (PH).

MW (kDa)	Fraction	MMP-1 Inhibition (µg OAE/mg Protein)	DPPH^●^ Inhibition (µg AAE/mg Protein)
<1 kDa	PH-I	6.2 ± 0.1 ^a^	3.5 ± 0.1 ^a^
1–3 kDa	PH-II	3.4 ± 0.2 ^b^	2.3 ± 0.0 ^b^
3–10 kDa	PH-III	1.3 ± 0.6 ^c^	1.8 ± 0.0 ^c^
>10 kDa	PH-IV	0 ± 0.9 ^d^	1.2 ± 0.1 ^d^

Note: PH = *A. domesticus* protein hydrolysate, MMP = matrix metalloproteinase, DPPH^●^ = 2,2-diphenyl-1-picrylhydrazyl radical. The MMP-1 inhibitory effect s expressed in micrograms of oleanolic acid equivalent per one milligram of protein (ug OAE/mg protein). The DPPH^●^ scavenging capability is expressed in micrograms of ascorbic acid equivalent per one milligram of protein (ug AAE/mg protein). The lowercase letters (a, b, c, and d) indicate significant variability between the samples. Statistical significance was analyzed using a one-way ANOVA followed by a post hoc Tukey test (*p* < 0.05).

**Table 7:** The amino acid sequence of selected peptide fractions from *A. domesticus* protein hydrolysate (PH) with potent activity against skin aging.

Fraction no.	Amino Acid Sequence	Length	Mass	ALC Score
99	AVTKADPYTDQ	11	1208	98
	AENQRVSFD	9	1064	97
	YLGGEGHNLQEH	12	1353	96
	SPLPKY	6	703	96
	EAKAAASAPVALHKAK	16	1562	96
	NGEPVYHP	8	911	95
106	TVMELNDLVKAF	12	1379	98
	FGGEAKDYSQ	10	1100	97
	WAPDLPGL	8	867	97
	FGSQDLSK	8	880	97
	AENQRVSFD	9	1064	96
	FGGEAKDY	8	885	95
	TGTVVSDKMD	10	1051	95
	AAAPAAPAAD	10	824	95
113	VPLLGPW	7	780	99
	VGTLGHVD	8	796	97
	YVAGAEGPQ	9	890	95

Note: A = alanine, R = arginine, N = asparagine, D = aspartic acid, C = cysteine, Q = glutamine, E = Glutamic acid, G = glycine, H = histidine, I = isoleucine, L = leucine, K = lysine, M = methionine, F = phenylalanine, P = proline, S = serine, T = threonine, W = tryptophan, Y = tyrosine, V = valine, and ALC = average local confidence.

## Data Availability

Data are contained within the article.

## Abbreviations

**Abbreviations**	**Definitions**
AA	Ascorbic acid
AAE	Ascorbic acid equivalent
ALC	Average local confidence
Ala	Alanine
AP-1	Activator protein 1
Arg	Arginine
Asn	Asparagine
Asp	Aspartic acid
CCD	Central composite design
CE	Crude extract
Cys	Cysteine
DEX	Dexamethasone
DH	Degree of hydrolysis
DPPH	2,2-diphenyl-1-picrylhydrazyl
E/S	Enzyme-substrate concentration
ECM	Extracellular matrix
GAPDH	Glyceraldehyde 3-phosphate dehydrogenase
Glu	Glutamic acid
Gln	Glutamine
Gly	Glycine
HaCaT	Immortalized human keratinocyte
His	Histidine
IC_20_	Concentration reducing cell viability by 20%
IC_50_	Half-maximal inhibitory concentration
Ile	Isoluecine
kDa	Kilodaltan
Lue	Luesine
Lys	Lysine
NF-κB	Nuclear factor kappa B
MAPKs	Mitogen-activated protein kinases
Met	Methionine
MMPs	Matrix metalloproteinases
MW	Molecular weight
OA	Oleanolic acid
OAE	Oleanolic acid equivalent
PC	Protein concentration
PH	Protein hydrolysate
Phe	Phenylalanine
Pro	Proline
RSM	Response surface methodology
Ser	Serine
Smad	Suppressor of mothers against decapentaplegic
TE	Trolox
TGF	Transforming growth factor
Thr	Threonine
Trp	Trptophan
Tyr	Tyrosine
UV	Ultraviolet
Val	Valine
VC	Vehicle control
